# Household smoking impact on the oral health of 5- to 7-years-old children

**DOI:** 10.1186/s12903-023-03715-3

**Published:** 2023-12-19

**Authors:** Abla Arafa

**Affiliations:** 1 Department of Pediatric Dentistry and Orthodontics, Faculty of Oral and Dental Medicine, Misr International University, 28, Cairo, Egypt; 2 Department of Preventive Dentistry, College of Dentistry, Umm AlQura University, Makka, Saudi Arabia

**Keywords:** Passive smoking, Children, Cotinine, Dental caries, Tooth hypomineralization

## Abstract

**Background:**

Children’s exposure to secondhand smoke, particularly by their parents, could adversely affect their oral health. Thereby, this study aimed to assess the oral health status of children subjected to household smoking and the impact of smoking patterns on the severity of oral health deterioration.

**Methods:**

A total of 210 healthy children were enrolled in this case-control study and allocated into children subjected to household smoking (HS) and control groups. Participants’ guardians were asked to complete a questionnaire regarding sociodemographic characteristics and parental smoking habits. All participants were subjected to clinical dental examination to assess dental caries (ICDAS), hypomineralized primary molars (HSPM), and gingival status (GI). Stimulated saliva samples were collected to assess saliva composition and characteristics. Urine samples were collected and analyzed for cotinine concentration. Data were analyzed using SPSS (v.25) software at a test value of *p* ≤ 0.05. The t-student test was used to find significant differences between participants’ age, gingival index score, saliva pH, flow rate, sIgA, and cotinine level. The Chi-square test was used to test for the significance of parental employment, number of rooms, gender, sweets consumption, brushing frequency, and HMPM. The correspondence analysis was used to test for significance of parents’ levels of education, type of house ventilation, ICDAS score, smoking form, frequency, and smoking pattern. The correlation between cotinine level and sIgA was tested for association using Bivariate correlation test.

**Results:**

The HS group showed a significantly increased risk for dental caries (*p* < 0.000), HSPM lesions (*p* = 0.007), and GI score (*p* < 0.000). A significant reduction in salivary flow rate, saliva pH, and sIgA were evident in HS group (*p* < 0.000). Parental consumption of more than 20 cigarettes/day was accompanied by increased dental caries activity (*p* < 0.000) and higher risk for increased severity of gingival inflammation (*p* < 0.000) of children in the HS group. Children of parents who smoke cigarettes and use the hubble/bubble anywhere in the house found to have greater distribution of HSPM (*p* < 0.000). Reduced sIgA values were found to be significantly associated with increased cotinine concentrations in HS children (*p* < 0.000).

**Conclusions:**

Frequent exposure to household smoking could be associated with an increased risk of dental caries progression, enamel hypomineralization, gingival inflammation, and saliva characteristics changes in children.

**Supplementary Information:**

The online version contains supplementary material available at 10.1186/s12903-023-03715-3.

## Introduction

Children’s exposure to secondhand smoke in their homes represents a major public health challenge. According to the key facts of tobacco released by the WHO, the global prevalence of adult smoking in 2020 was 32.6 and 6.5% among males and females respectively [1]. The WHO report about the Eastern Mediterranean Region indicated that the prevalence of tobacco consumption in Saudi Arabia was 14.9% by young adults and 12.2% by adults where prevalence of smoking was found higher among the males compared to the females at 25.34 and 1.91% respectively [2, 3]. Second-hand smoking, involuntary smoking, passive smoking, and exposure to environmental tobacco smoke (ETS), are synonyms for the state of inhaling smoke exhaled by another individual. Third-hand smoke describes the residual nicotine and other chemicals left on indoor surfaces or remaining on clothes, carpets, and furniture following individual smoking. Children have been found to be more vulnerable to the impact of household smoking owing to their higher breathing rate per body weight, organ immaturity, and relatively smaller body size compared to adults [4].

Children were found to be the most affected group by passive smoking among nonsmoking individuals. More than one-third of children live with one or more smoker family members [5]. In addition, as a result of the closure of schools and afterschool clubs following the COVID-19 pandemic, more children spent additional time at home, thus intensifying their exposure to passive parental household smoking which in turn could leave its adverse short- and long- term impact on the dental status of children and oral health [6].

The secondhand smoking has an adverse impairment on the health of nonsmoker regardless of their age group. Children exposed to parental secondhand smoke were reported to be at higher risk for complicated health and behaviour problems [7]. Tobacco smoke contains a vast variety of chemical agents, such as nicotine that could adversely affect the oral health of passive smokers [8]. Cotinine, a nicotine metabolite; could be used as a possible monitoring biochemical marker for smoking behaviour since it can be detected in various body fluids and secretions such as saliva and urine [9].

Some researchers consider exposure to tobacco smoke even if occurred indirectly, to be a possible factor of dental impairments and dental caries progression [10]. Reduced alveolar bone density, and aggressive loss of the supporting periodontium can also be observed as a serious consequences of negative smoking with increased incidence of gingival pigmentation in children and adolescents [11]. In addition, a possible association between parental smoking and hypomineralization of second primary molars HSPM has also been suggested [12, 13]. Alterations of the oral microbiome, reduction in the salivary flow rate and changes in saliva composition have also been reported to be associated with increased exposure to tobacco smoke particularly if household [14].

Several studies have focused on the effect of tobacco smoking and the impact of secondhand smoke on oral health status; however, scanty studies have investigated the possible association between household smoking and the oral health status and salivary composition of children. The null hypothesis stated that no difference can be found in the oral health status and salivary composition in children exposed to household smoke compared to children not exposed to household smoke. Therefore, the present study aimed to assess the oral health condition of children subjected to household smoking and the impact of smoking patterns on the severity of oral conditions.

## Materials and methods

### Ethics approval and consent to participate

The ethical approval of this study was obtained from the local institutional scientific research ethical committee, Umm AlQura University, KSA (HAPO-02-K-012-1412), and conducted according to the Declaration of Helsinki. A written informed consent was received from each participant’s guardian following a comprehensive explanation of the research methodology and any anticipated inconveniences.

### Study design

The study sample of this cross sectional study consisted of healthy children aged 5- to 7-years-old attending the outpatient clinics at the Faculty of Dentistry, Umm AlQura University, Saudi Arabia; during the period from May to September, 2022.

For sample size determination, the risk of dental caries progression was 14% with anticipated increase of 30% for children not exposed to household smoking and children exposed to household smoking respectively [15]. To acquire 80% study power with a two-sided α equal to 0.05, a minimum of 104 participants per group were needed.

Participating children were matched between case and control groups regarding age, gender, parental education and social class to guard against the confounder effect.

The administered questionnaire tool was created based on formerly validated questionnaire [16, 17]. The questionnaire consisted of 26 closed questions regarding their sociodemographic characteristics and parental smoking habits. The selection criteria for children to be enrolled in the present study were as follows; If a child had been subjected to regular household smoking since birth regardless the amount or the frequency of smoking; he/she was grouped into the household smoking HS group (*n* = 105). The control group consisted of children who had not been exposed to household smoking by either parents since birth (*n* = 105). Children who suffered from any systemic illness or reported chronic use of medication were not included in the study. The enrolled children were only whom parent/legal guardian accepted voluntary participation in this study.

### Clinical procedure

All children were examined clinically by the same examiner, a consultant of Pediatric Dentistry; using a sterile disposable diagnostic kit for each participant while sitting on a regular chair. Each diagnostic kit consisted of plane mouth mirror, sickle explorer No.23, spoon excavator, and tweezer.

The examiner received a training process followed by calibration exercise using photographic slides of clinical cases of each criterion of ICDAS and HSPM assessment to reach acceptable level of agreement and consistency of findings. The data of 18 patients were recorded at the baseline then re-calibrated after one-week interval [18]. Intra-examiner reliability was evaluated using SPSS program where almost perfect agreement level was achieved (Kappa = 85.9%).

Dental caries assessment was carried out according to the International Caries Detection and Assessment System (ICDAS) criteria; code 1 was assigned when the enamel surface showed early visual changes confined to pits and fissures following air drying, code 2 was assigned for: apparent visual changes in enamel, code 3 was assigned for: demarked breakdown in opaque enamel surface without evidence of dentinal effect, code 4 was assigned when: dark shadow became evident in dentin, code 5 was assigned for: distinct cavitated lesion in dentin, and code 6 was assigned for: extensive cavity involving more than half of the dentin surface [19]. According to the highest ICDAS score, the participants were divided as follows: low caries activity involving only enamel (codes 1–3), moderate caries activity involving enamel and dentine (code 4), and high caries activity involving cavitated (codes 5 and 6) caries lesions [20].

Assessment of the hypomineralized second primary molar (HSPM) was carried out using the MIH/HSPM index following the European Academy of Paediatric Dentistry (EAPD) molar incisor hypomineralization (MIH) diagnostic criteria modified for HSPM assessment. The tooth was considered hypomineralized if any of the following characteristics were observed; demarked opacities of enamel, post eruptive enamel breakdown (PEB), atypical carious lesions or restorations or extractions that do not match the dental caries pattern of the child [21, 22].

The gingival Index (GI) was used to assess gingival health status [23]. The gingival inflammation severity was scored on a scale from 0 to 3 following gingival inspection per surface according to the following criteria; score 0; no signs of inflammation, score 1; mild inflammation, slight redness, slight oedema, probing with a blunt probe did not elicit bleeding, score 2; moderate inflammation, oedema, redness, glazing, with swollen marginal gingiva and bleeding accompanying probing using a blunt explorer, and score 3; severe inflammation, marked redness and oedema, spontaneous bleeding and/or ulceration. The tooth score was calculated by dividing the sum of all surface scores, by four. Only fully erupted teeth were examined. The individual GI score was calculated for every participant via dividing the sum of all assessed teeth divided by the total number of examined teeth. According to the GI score, the participants were categorized to; score 0 for not inflamed gingiva, score 0.1–1 for mild inflammation, score 1.1–2 for moderate gingival inflammation and score 2.1–3 for severe gingival inflammation [24].

### Assessment of saliva samples

The collection of saliva samples was carried out using the masticatory stimulated saliva method between 9:00 AM and 12:00 PM, 2 hours after breakfast. Each participant was kindly guided to set motionless in an upright position and swallow (time of start), then was asked to chew on a piece of paraffin wax for 30 sec. and lean his/her head foreword to collect the stimulated saliva volume over 5 min. in a sterile graduated container. The saliva flow rate was calculated by dividing the volume of the collected saliva over time (ml/min) [25]. Immediately following the collection of saliva samples, saliva pH was measured via a precalibrated digital pH meter (AD1000, ADWA Instruments Kft., Szeged, Hungary). Collected saliva samples were stored at − 80 °C (Thermo Fisher Scientific LLC Model No. UXF40086D62, Asheville, NC USA) until utilized for analysis of secretory IgA levels. At the time of testing, the samples were brought to room temperature and then placed in the centrifugation machine (Multifuge X1R Centrifuge, USA) at 3000 rpm for 15 min. The supernatant from each sample was used to measure the level of sIgA (μg/ml). A qualified lab technician under the supervision of specialist of microbiology ran the ELISA tests for sIgA using DRG® IgA Salivary ELISA (SLV-4636, DRG International, Inc. USA) following the manufacturer’s instructions. The sIgA kit was provided with IgA standards S0 – S4 (5 vials, 1 mL each) and control reagents (1 vial, 1 mL) ready for use. Concentration of Control is Lot-specific. The standard concentrations are 1000 times lower than the values reported in the reference range because the samples are diluted 1:1000 while the standards are not. The Standard concentrations to be used for calculation were: S0 S1 S2 S3 S4 at 0, 6.9, 62, 132, and 400 μg/mL. The controls should be treated as unknowns and values determined as the test procedure conducted.

### Assessment of cotinine level

Each participant was provided with a 120 ml clean plastic container to collect a urine sample at the end of the clinical examination visit. Collected urine samples were coded and stored at − 20 °C refrigerator (Thermo Fisher Scientific LLC Model No. UXF40086D62, Asheville, NC USA) until analysis for cotinine (ng/ml) using DRG® Cotinine (Urine) ELISA (EIA-1377, DRG International, Inc. USA) following the manufacturer’s instructions. The calibrators were included when the assay was conducted. The negative calibrator consisted of I mL of urine matrix negative for cotinine. The positive calibrators consisted of 1 mL of urine matrix containing 50, 500 (Cut-off Calibrator), and 5000 ng/mL cotinine. The negative control should have greater absorbance than the cut-off while the positive control should have lower absorbance than the cut-off calibrator. Reading the stopped assay was carried within 15 minutes at 450 nm.

The concentrations of sIgA in saliva and cotinine in urine were read using SPECTROstar®^*Nano*^ (Nano microplate reader, BMG LABTECH, Germany). All data were displayed using Multi-user Reader Control and MARS data analysis software (BMG LABTECH, Germany).

### Statistical analysis

The statistical analysis was carried out using SPSS software version 25 (SPSS Inc., IBM Crop, Armonk, NY, USA) to test for significance at *p* ≤ 0.05. The collected data were explored for normality using Kolmogorov Smirnov statistical test (sample size ≥50), and the data were found approximate normal (kurtosis excess between − 1 and + 1). The z-test was applied for normality where at absolute z-value ±3.29 (medium-sized sample 50 ≤ *n* = 105/gp ≤300), normal distribution of the sample was detected [26, 27]. Student’s t-test was used to find significant differences between continuous values, such as participants’ age, gingival index score, saliva pH, flow rate, sIgA level, and cotinine level. The chi-square test was used to test for the significance of categorical and binomial data, such as parental employment, number of rooms, gender, sweets consumption, brushing frequency, and HMPM. The correspondence analysis was used to test for significance if one of variables has more than two values as with the variables of parents’ levels of education, type of house ventilation, ICDAS score, smoking form, frequency of smoking per day, and smoking pattern. The correlation between cotinine levels in urine and sIgA was tested for association using Bivariate Pearson correlation coefficient test.

## Results

**Fig. 1:**
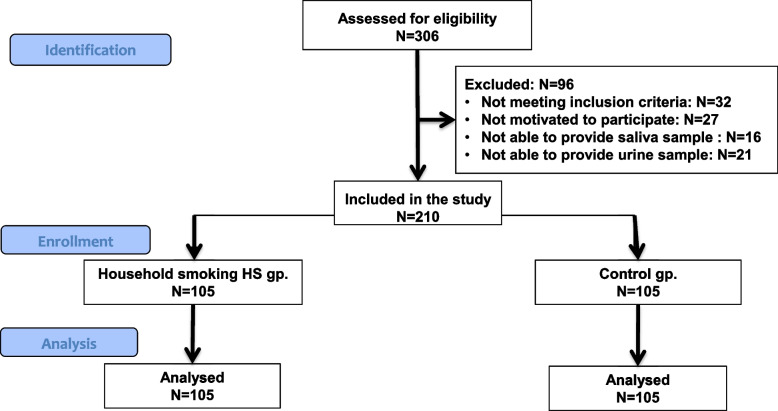
STROBE Flow diagram illustrating the process of participants’ enrollment in this study

A total of 210 participants were included in this study of the 306 patients identified for eligibility. Patients who didn’t meet the inclusion criteria, or not motivated to be enrolled, or were not able to provide saliva/urine samples were not included in the study (Fig. 1).

### Sociodemographic characteristics

The assessment of sociodemographic characteristics revealed that no statistically significant difference can be identified between the HS group and the controls regarding the level of parental education, employment, number of children in family, number of existing rooms and presence or absence of adequate ventilation (Table 1).

**Table 1:** Sociodemographic characteristics of the participants

Variable			Control (*n* = 105)	HS (*n* = 105)	*p*-value
***Level of education***	Mothers	Illiterate	28(13.12%)^a^	24(11.25%)^a^	0.347
		High school	31(14.37%)^a^	42(20%)^a^	
		Bachelor	46(22.5%)^a^	39(18.75%)^a^	
	Fathers	Illiterate	25(11.87%)^a^	24(11.25%)^a^	0.586
		High school	39(18.75%)^a^	46(21.88%)^a^	
		Bachelor	41(19.37%)^a^	35(16.88%)^a^	
***Parental employment***		One parent	79(37.5%)^a^	68(32.5%)^a^	0.132
		Both	26(12.5%)^a^	37(17.5%)^a^	
***Number of children***			2.35 ± 0.73^a^	2.51 ± 0.71^a^	0.109
***Number of rooms***		≤ 2 rooms	88(41.87%)^a^	92(43.75%)^a^	0.555
		≥ 3 rooms	17(8.12%)^a^	13(6.25%)^a^	
***Ventilation***		None	13(6.19%)^a^	6(2.86%)^a^	0.177
		Ventilator	66(31.43%)^a^	76(36.19%)^a^	
		AC	26(12.83%)^a^	23(10.95%)^a^	

Different lower case superscripts in the same row indicate significance. Letter "b" superscript indicates group of statistical significant difference compared to group/s indicated with letter "a" superscript

### Clinical characteristics

The comparison between the HS and the control group revealed the absence of gender predilection or any statistically significant difference in sweets consumption habits or brushing frequency. The HS group presented with statistically significant higher score of ICDAS, HSPM and GI (Table 2). Smoking frequency higher than 20 cigarettes/day showed a significant association with moderate to high caries activity while smoking anywhere in the house was found to be significantly associated with increased caries severity (Table 3). Smoking cigarette only and smoking anywhere in the house showed limited impact on occurrence of HSPM and found associated with absence of HSPM (Table 4). Smoking frequency more than 20 cigarettes/day showed statistical significant association with moderate severity of gingival inflammation in HS group (Table 5).

**Table 2:** Clinical characteristics of the participants

Variable		Control (*n* = 105)	HS (*n* = 105)	*p*-value
***Gender***	Female	55 (26.19%)^a^	52 (24.76%)^a^	0.783
	Male	50 (23.8%)^a^	53 (25.23%)^a^	
***Age***		6.07 ± 0.97^a^	6.11 ± 0.86^a^	0.752
***Sweets consumption***	≤1/day	71(50.9%)^a^	68(49.1%)^a^	0.771
	≥2/day	34(48.1%)^a^	37(51.9%)^a^	
***Brushing frequency***	<2/day	32(57.1%)^a^	23(42.9%)^a^	0.209
	≥2/day	73(47.5%)^a^	82(52.5%)^a^	
***ICDAS***	No caries	30(14.29%)^a^	20(9.52%)^a^	0.000
	Low(1–3)	54(25.71%)^b^	22(10.48%)^a^	
	Moderate(4)	8(3.81%)^a^	17(8.09%)^a^	
	High(5,6)	13(6.19%)^a^	46(20.48%)^b^	
***HSPM***	Yes	9(4.38%)^a^	24(11.25%)^b^	0.007
	No	96(45.63%)^a^	81(38.75%)^a^	
***GI***		0.98 ± 0.43^a^	1.43 ± 0.44^b^	0.000
***Flow rate(mL/min)***		1.31 ± 0.33^a^	0.96 ± 0.19^b^	0.000
***pH***		6.48 ± 0.65^a^	6.01 ± 0.61^b^	0.000
***Cotinine(ng/ml)***		1.01 ± 0.19^a^	2.11 ± 0.29^b^	0.000
***sIgA(μg/ml)***		170.17 ± 32.97^a^	52.55 ± 26.93^b^	0.000

Different lower case superscripts in the same row indicate significance. Letter "b" superscript indicates group of statistical significant difference compared to group/s indicated with letter "a" superscript

**Table 3:** Household smoking characteristics along different levels of caries activity

Variable	No caries (*n* = 22)	Low (*n* = 25)	Moderate (*n* = 32)	High (*n* = 26)	*p*-value
***Smoking form***					
Cigarette	14(13.33%)^a^	21(20%)^a^	26(24.76%)^a^	24(22.8%)^a^	0.261
Hubble/bubble	4(3.8%)^a^	3(2.85%)^a^	4(3.8%)^a^	1(0.95%)^a^	
Both	4(3.8%)^a^	1(0.95%)^a^	2(1.9%)^a^	1(0.95%)^a^	
***Smoking frequency/day***					
<10 cigarettes/day	12(11.43%)^b^	5(4.76%)^a^	2(1.9%)^a^	2(1.9%)^a^	0.000
10–20 cigarettes/day	5(4.76%)^a^	13(12.38%)^a^	8(7.62%)^a^	4(3.81%)^a^	
> 20 cigarettes/day	5(4.76%)^a^	7(6.66%)^a^	22(20.95%)^b^	20(19.05%)^b^	
***Smoking pattern***					
Specific room	8(7.62%)^a^	8(7.62%)^a^	4(3.81%)^a^	0(0%)^a^	0.003
Anywhere in house	14(13.33%)^a^	17(16.19%)^a^	28(26.66%)^a^	26(24.76%)^b^	

Different lower case superscripts in the same row indicate significance. Letter "b" superscript indicates group of statistical significant difference compared to group/s indicated with letter "a" superscript

**Table 4:** Household smoking characteristics along the distribution of HSPM

Variable	Present (*n* = 24)	Absent (*n* = 81)	*p*-value
***Smoking form***			
Cigarette	11(24.76%)^a^	74(22.8%)^b^	0.000
Hubble/bubble	6(5.71%)^a^	6(5.71%)^a^	
Both	7(1.9%)^b^	1(0.95%)^a^	
***Smoking frequency/day***			
<10 cigarettes/day	6(5.71%)^a^	15(14.29%)^a^	0.297
10–20 cigarettes/day	9(8.57%)^a^	21(20.0%)^a^	
> 20 cigarettes/day	9(8.57%)^a^	45(42.57%)^a^	
***Smoking pattern***			
Specific room	9(8.57%)^b^	11(24.76%)^a^	0.009
Anywhere in house	15(14.29%)^a^	70(66.67%)^b^	

Different lower case superscripts in the same row indicate significance. Letter "b" superscript indicates group of statistical significant difference compared to group/s indicated with letter "a" superscript

**Table 5:** Household smoking characteristics along the severity of gingival inflammation

Variable	Not inflamed (*n* = 9)	Low (*n* = 25)	Moderate (*n* = 56)	Severe (*n* = 15)	*p*-value
***Smoking form***					
Cigarette	8(7.62%)^a^	18(17.14%)^a^	45(42.57%)^a^	14(13.33%)^a^	0.560
Hubble/bubble	0(0.0%)^a^	5(4.76%)^a^	6(5.71%)^a^	1(0.95%)^a^	
Both	1(0.95%)^a^	2(1.90%)^a^	5(4.76%)^b^	0(0.0%)^a^	
***Smoking frequency/day***					
<10 cigarettes/day	4(3.81%)^a^	5(4.76%)^a^	8(7.62%)^a^	4(3.81%)^a^	0.001
10–20 cigarettes/day	5(4.76%)^a^	12(11.43%)^a^	10(9.52%)^a^	3(2.86%)^a^	
> 20 cigarettes/day	0(0.0%)^a^	8(7.62%)^a^	38(36.19%)^b^	8(7.62%)^a^	
***Smoking pattern***					
Specific room	9(8.57%)^a^	21(20.0%)^a^	41(39.05%)^a^	14(13.33%)^a^	0.109
Anywhere in house	0(0.0%)^a^	4(3.81%)^a^	15(14.29%)^a^	1(0.95%)^b^	

Different lower case superscripts in the same row indicate significance. Letter "b" superscript indicates group of statistical significant difference compared to group/s indicated with letter "a" superscript

### Biological variables

**Fig. 2:**
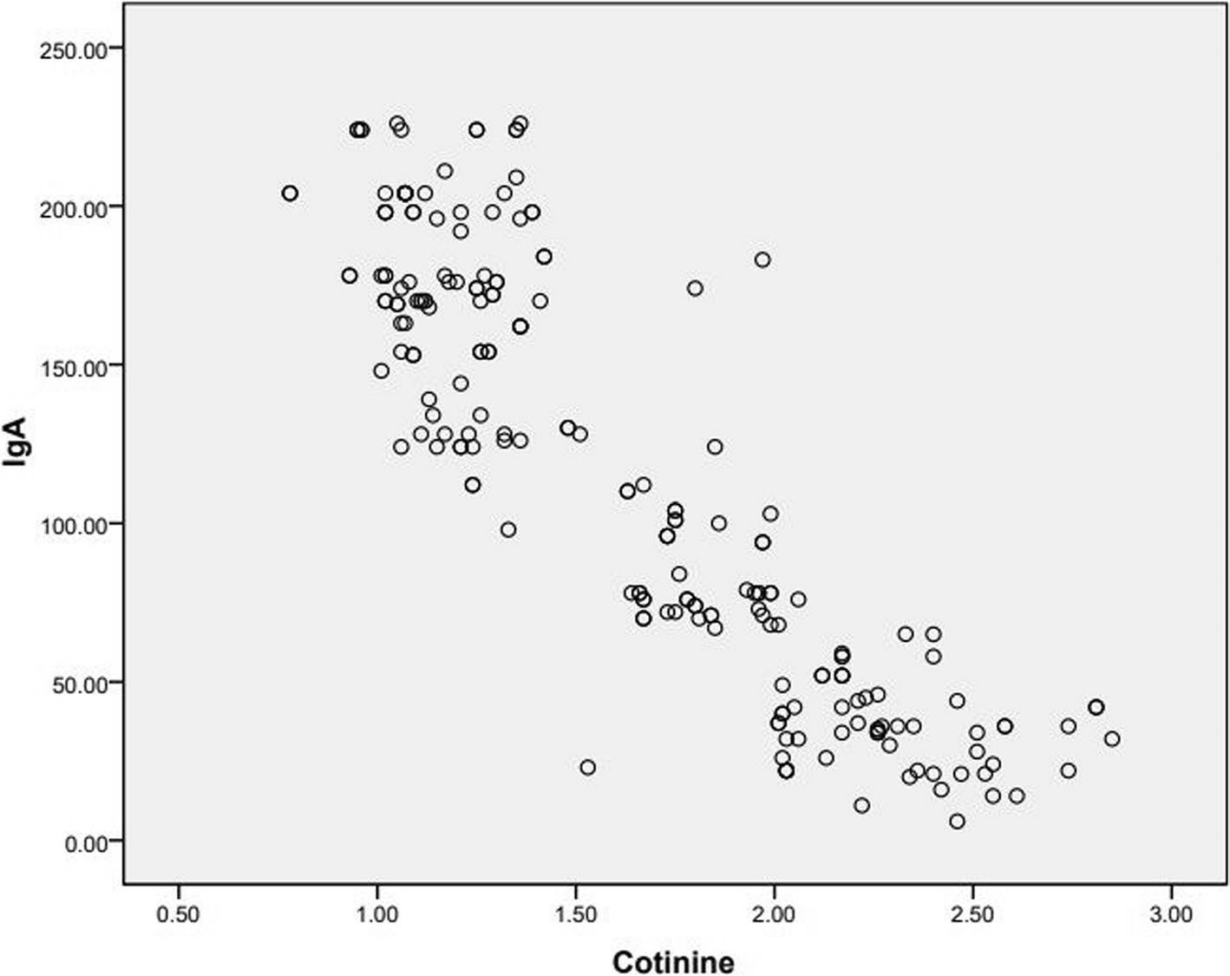
Scatter diagram depicting a statistically significant inverse relationship between the level of cotinine in urine (ng/ml) and salivary immunoglobulin A (μg/ml). The increase in cotinine level found to be inversely associated with reduced sIgA

Children subjected to household smoking showed statistically significant lower mean values of saliva flow rate, pH, and sIgA with higher mean value of cotinine in urine (Table 2). An inverse relationship has been detected between the mean value of cotinine and sIgA (*r* = − 0.888, *p* < 0.000). The increase in cotinine level in urine denoting exposure to tobacco smoke found to be associated with statistically significant with reduced sIgA in saliva (Fig. 2).

## Discussion

The results of the current study affirm the harmful impact of parental household smoking on their children oral health. Children exposed to parental household smoking since birth found to be at increased risk of dental caries, gingival inflammation and hypomineralization of second deciduous molars. Pattern and frequency of parental smoking found to be associated with high dental caries activity.

During- and after the COVID-19 pandemic lockdown, increased children’s exposure to tobacco smoke was reported. This could be attributed to reduced children’s outdoor activities and being kept home from school with increased contact with their parents who were also found to have changed smoking habits with limited access to proper dental care [28–30].

All tobacco products contain “nicotine” as the main component of their chemical constituents. Most of the nicotine ingress becomes metabolized to cotinine via liver enzymes. Cotinine has been selected as a biomarker for estimating exposure to secondhand smoking since it has a longer half-life (t_1/2_) of an average of 16 hours compared to 2 hours for nicotine. Thus, cotinine concentrations are more stable throughout the day and can be detected in blood, urine, or saliva. However, urine concentrations of cotinine possess four- to six folds the average concentration than that of saliva or blood. Accordingly, urine can be considered a noninvasive, highly sensitive matrix to detect even minimal concentrations caused by tobacco smoke exposure [31]. The number of cigarettes consumed by the smoker was found to have an association with the cotinine mean value in the urine of passive smokers [11]. Biofluids of children exposed to secondhand smoke were found to have higher cotinine concentrations compared to adults who contributed to higher ventilation per body mass of children who frequently remained close to the smoking parents along with slower cotinine metabolism in children [32].

The use of the International Caries Detection and Assessment System (ICDAS) as a caries experience assessment tool has a high sensitivity and specificity. Despite the slightly extended clinical examination time when using the ICDAS index compared to the World Health Organization (WHO) criteria, the ICDAS allows early detection of initial noncavitated caries lesions which are dominate in children [33]. Early diagnosis of incipient caries shifted the focus to prevention strategies and control of disease progression [34, 35].

The assessment of dental caries experiences using the ICDAS score in the present study revealed an increase in caries activity affecting children who were exposed to household smoking and was found significantly linked with increased smoking frequency and parents smoking anywhere in the house. Similarly, it has been reported that a high prevalence of dental caries could be associated with children exposure to secondhand smoke where the increase in dental caries risk was found to reach approximately one and a half fold compared to children not exposed to secondhand smoke [15, 16]. This could be attributed to nicotine’s impact on encouraging cariogenic bacteria proliferation and attachment to tooth surface, especially in children affected by caries-forming microorganisms from their caregivers [36–38]. In addition, it has been reported that nicotine exposure increases biofilm formation and the precipitation of extracellular polysaccharides which increase in thickness proportionally with the increase in nicotine exposure [39]. Furthermore, children subjected to passive tobacco smoke were found to suffer from repeated upper respiratory tract infections which could be accompanied by mouth breathing, reduced salivary flow rate, and diminished saliva protective factors [40]. As found in the present study, the increased caries activity was not only accompanied by a reduced salivary flow rate; but also by changes in saliva composition in terms of a reduction in the sIgA mean value which was found to be inversely proportional to the cotinine mean value.

The results of the present study revealed increased susceptibility to HSPM in children subjected to household smoking where limited impact of smoking form and pattern has been identified to affect the distribution of HSPM. Hypomineralization of the second primary molar (HSPM) describes a qualitative defect affecting tooth enamel that appears during development as well as demarcated opacity with possible enamel breakdown affecting one- to four-primary second molars [31]. The affected enamel was found to be porous and more liable to break down soon post-tooth eruption into the oral cavity. Thus, it could be accompanied by increased susceptibility to caries development [41]. Strict adherence to the adapted diagnostic criteria of the MIH/HSPM index would aid in recording the actual extent of the HSPM spectrum and could provide insight into the progression of HSPM over time [42]. Similar to the results of this study, it has been reported that parental smoking could play a critical role in the development of HSPM [43]. HSPMs could arise from interacting multifactorial causes. Repeated upper respiratory tract infections commonly occurring in children subjected to secondhand smoke were reported as a possible risk for developing HSPM [9]. Systemic fever and elevated body temperature could affect ameloblasts, resulting in serious interference with enamel formation and consequently causing hypomineralization. It has been reported that exposure to smoke could influence ameloblasts causing alterations in their function and affecting hard tissue mineralization of developing teeth [44].

The first years of life represents the most critical period for enamel defects to occur, as this is the period of dental organ development and maturation. Insult exposure during this period could induce second primary molar effects [42]. It has been suggested that the assessment of HSPM should be carried out optimally around the age of 5 years since in the younger age group, the impact of synoptic demolition veiling the original defect was found to be less likely to occur [41]. In addition, in the older age range (above the age of eight-year-old); dental caries could interfere with the accurate identification of HSPM-affected teeth with an increased possibility for restoration placement concealing the original defect [45]. Accordingly, children included in this study were from five- to 7 years old. HSPM yield the patient highly prone to develop more severe carious lesions [46].

The results of this study also revealed that youth exposed to household smoking presented with an increased risk of developing gingival inflammation particularly if smoking frequency exceeded 20 cigarette per day. Chemical products from smoking could yield oedema and diffuse inflammation by eliciting the activity of inflammatory agents and local vasoconstriction. Systemically, as found in the present study; these products were able to inhibit in saliva IgA to a mean level proportional to the degree of exposure to tobacco smoke. The effect of the host immune responses in terms of decreases in the phagocytic activities of neutrophils and macrophages, and suppression of T-helper cell function were found to be associated with increased exposure to tobacco smoke. This could be associated with gingival and periodontal effects characterized by a marked decrease in alveolar bone density and eventual loss of teeth. Products from cigarette smoke are found to slow the healing of wounds and nicotine residues prohibit cellular proliferation and osteoblastic and fibroblastic productivity [47]. In addition, mouth breathing and mouth dryness accompanied by frequent respiratory tract infections were found to be an additional reason behind gingival and periodontal effects [39].

Early examination of children subjected to household smoking would not only aid in the early detection of dental and oral problems but also augment the proper provision of requested dental care to avoid progressive complications. Dental teams concerned with offering professional dental care should be aware of the possible dental risks associated with children’s exposure to secondhand smoke. A comprehensive orientation of smokers particularly parents, regarding the oral and dental problems developed in young children as a result of tobacco smoke exposure could motivate them to quit, lessen or at least try to avoid the adverse effect on other individuals especially children.

The limitation of this study could arise from the restricted age range of children participated in the study. To assess the impact of household smoking on oral health of children, larger age interval had to be included and correlated to the intensified severity of oral and dental changes. In addition, the enrolled participants in this study were among the attendee of public facility while those reporting to the private sector were not included in this investigation, thus affecting the generalization of the concluded outcomes.

## Conclusion

Under the limitations of the present study, the following can be concluded;

Children exposure to household smoking could induce serious changes in saliva composition and yield exposed individuals at higher risk to develop dental caries, hypomineralization, and gingival inflammation. The frequency and pattern of parental smoking could have an association with increased severity of dental caries progression, hypomineralization of deciduous molars, and gingival inflammation in affected children.

### Supplementary Information

**Additional file 1.**

## Data Availability

The datasets used and/or analysed during the current study available from the corresponding author on reasonable request.
