# Supplementary material for: Household smoking impact on the oral health of 5- to 7-years-old children
Source: BMC Oral Health. 2023 Dec 19;23:1028. doi: 10.1186/s12903-023-03715-3 (PMC10731723; doi:10.1186/s12903-023-03715-3)
Supplement: Supplementary file 1 — Additional file 1. [file 12903_2023_3715_MOESM1_ESM.pdf]

<table>
<tr><td>Code</td><td></td><td>1.Date of Birth</td><td></td><td>2.Gender</td><td></td></tr>
<tr><td>3.Child school grading</td><td colspan="5">□Non educated □ 1st grade □ 2nd grade □3rd grade □4th grade □5th grade</td></tr>
<tr><td>4.Phone number</td><td colspan="2"></td><td>5.Residence</td><td colspan="2"></td></tr>
<tr><td>6.Mother occupation</td><td></td><td>7.Mother education level</td><td colspan="3">□ Illiterate
□ High school
□ Bachelor</td></tr>
<tr><td>8.Father occupation</td><td></td><td>9.Father education level</td><td colspan="3">□ Illiterate
□ High school
□ Bachelor</td></tr>
<tr><td colspan="2">10.Number of children in house</td><td>11.Kitchen type</td><td colspan="3">□Closed kitchen □Open kitchen</td></tr>
<tr><td colspan="2">12.NUMBER OF ROOMS IN HOUSE</td><td colspan="4"></td></tr>
<tr><td>13.VENTILATION IN HOUSE</td><td colspan="5">□AIR CONDITION □FAN □NATURAL VENTILATION</td></tr>
<tr><td colspan="6">14.CHILD COMPLAIN</td></tr>
<tr><td colspan="6">Health problems suffered by child
□ Sneezing
□ Coughing
□ Asthmatic attacks
□ Recurrent respiratory infections NO of respiratory tract infections /year……………..
□ Otitis media
□ Cardiovasular problems……………………….
□ Gastrointestinal problems…………………..
□ Gentiurinary disorders………………………….
□ Neuropshycatric disordes…………………….
□ Others ………………………………………………..
If any state disorder beside it-------------------------------</td></tr>
<tr><td>15.Father smokes</td><td>□ Yes
□ No</td><td>□ Cigg smoker
□ Hubble bubble smoker</td><td colspan="3">17.No of cigg /day □ <10cig/d
□10-20cig/d
□ >20cig/d
If hubble bubble, No of stones/day…..</td></tr>
<tr><td colspan="2">18.FATHER SMOKES SINCE</td><td colspan="4"></td></tr>
<tr><td>19.Mother smokes</td><td>□ Yes
□ no</td><td>□ Cigg smoker
□ Hubble bubble smoker</td><td colspan="3">21.No of cigg /day □ <10cig/d
□10-20cig/d
□ >20cig/d
If hubble bubble, No of stones/day………</td></tr>
<tr><td colspan="2">22.MOTHER SMOKES SINCE</td><td colspan="4"></td></tr>
<tr><td colspan="2">23.Smoking outside the house</td><td colspan="4">□YES □NO</td></tr>
<tr><td colspan="2">24.Smoking inside the house</td><td colspan="4">□YES □NO</td></tr>
<tr><td colspan="2">25.Smoking anywhere in the house</td><td colspan="4">□YES □NO</td></tr>
<tr><td colspan="2">26.Smoking in a specified room</td><td colspan="4">□YES □NO</td></tr>
</table>
